# And Artificial Intelligence Created the Polyps…

**DOI:** 10.1055/a-2904-9251

**Published:** 2026-07-08

**Authors:** Tommy Rizkala, Luca Di Stefano, Cesare Hassan

**Affiliations:** 1 Department of Biomedical Sciences 437807 Humanitas UniversityMilanItaly; 2 Department of GastroenterologyIRCCS Humanitas Research HospitalRozzanoItaly

## 

Colorectal cancer remains the third-most common malignancy and a leading cause of cancer death worldwide, and colonoscopy is the principal tool for interrupting the adenoma–carcinoma sequence. 1 The quality of that intervention depends heavily on the endoscopist, and the two skills that matter most—detecting lesions and characterizing them—are learned from images. Yet high-quality, well-annotated endoscopic images are surprisingly scarce. They are locked behind the legitimate but slow machinery of ethics approval, informed consent, and anonymization, and publicly available datasets remain limited and skewed toward common morphologies. This scarcity constrains both the human training that improves optical diagnosis 2 3 and the development of the artificial intelligence (AI) systems that now augment detection in routine practice. 4 5 The question of where the next generation of teaching images will come from is therefore neither abstract nor premature; it is a practical bottleneck for endoscopic education.

In this issue of *Endoscopy International Open* , Sodmann and colleagues offer a provocative answer: make the images. Building on more than 40 million frames from over 7,000 examinations across eight centers, the authors trained a latent diffusion model to generate high-resolution colorectal polyp images, then tested whether experienced endoscopists could tell synthetic from real. In an international, multicenter blinded reader study, 53 endoscopists from 14 countries classified 40 images, evenly split between real and AI-generated polyps. The results are striking: overall classification accuracy was 73%, with a sensitivity for identifying AI-generated images of only 66% and a corresponding specificity of 80% for real images. In a balanced forced-choice task, this 73% accuracy corresponds to a chance-corrected accuracy of just 46%, and readers reported significantly lower confidence when judging synthetic images. The free-text comments are perhaps the most telling: several participants admitted they were largely guessing.

Why does this matter? The authors are appropriately careful to frame their model as a tool for teaching rather than for augmenting AI training datasets, and that framing is where the contribution lands. If synthetic polyps are perceptually indistinguishable from real ones to expert eyes, they could supply an essentially unlimited, privacy-preserving pool of teaching material, decoupling endoscopic education from the consent-and-anonymization pipeline that rate-limits it. This is the same promise synthetic imaging has held out in radiology, where generative models offer a route to share data across institutions without exposing patient identity. 7 The radiology experience points to uses that map onto endoscopic training. Synthetic thorax radiographs with inserted lung nodules and perfect ground truth have been used in a reader study to pinpoint where radiologists systematically miss lesions 8 —a controlled curation of difficult cases no consecutive clinical series could guarantee. Diffusion models have likewise synthesized examples of underrepresented conditions, narrowing diagnostic gaps for rarer presentations across chest radiography, histopathology, and dermatology. 9 Reproducing that promise in endoscopy—with a single diffusion model rather than the patchwork of methods and resolutions used in earlier polyp-generation work—is a genuine methodological advance. The accompanying embedding analysis, showing that synthetic images occupy the same region of feature space as real polyps, yet remain distinct from their nearest training neighbors, guards against the obvious objection that the model is simply memorizing its inputs.

It is equally important to be clear about what the study does not show. Perceived realism is a necessary but not sufficient condition for educational value. The central endpoint here is whether experts can distinguish synthetic from real images, not whether trainees who learn from synthetic images become better at optical diagnosis or detect more adenomas in vivo. Those are different questions, and only the latter speaks to patient outcomes. The realism itself also warrants measured enthusiasm. Both image sets were manually curated for quality, the confidence rating was a coarse binary, and readers were given no instruction in the tell-tale artifacts of generated images. As the authors acknowledge, features such as sharpness and mucosal reflections may have driven the high sensitivity for real images, so the test may partly reflect image aesthetics rather than polyp authenticity. Most consequentially, the model reproduces the distribution of what it was trained on: it renders common morphologies well but cannot reliably produce the rare, atypical, and subtle lesions—including lateral spreading tumors—that are precisely the ones trainees most need to see and most often miss. This concern is not hypothetical: even mature computer-aided diagnosis systems show performance that varies by lesion location, 6 a reminder that representativeness, not just realism, determines whether image-based tools generalize. A teaching set that over-represents the easy cases risks instilling false confidence.

These caveats temper the immediate clinical implications without diminishing the direction of travel. For now, this is a proof of concept, not a teaching curriculum. The sensible next steps follow directly from the limitations: a prospective study testing whether endoscopists trained on synthetic images improve their optical diagnosis accuracy against a histological ground truth, ideally benchmarked against conventional image atlases; expansion of the generative model to encompass rare and difficult lesions; and incorporation of validated synthetic images into the structured competence frameworks that bodies such as the ESGE have already articulated for optical diagnosis. The ESGE curriculum is explicit about its building blocks—attending a validated training course built on a validated classification, computer-based and online learning, self-assessment of a minimum number of lesions with histological feedback (for diminutive colorectal lesions, at least 120), and online assessment modules with feedback to maintain competence 3 —and each is precisely the kind of standardized, image-hungry component a validated synthetic library could feed at scale. A further frontier, beyond the scope of this study, is whether synthetic images can responsibly augment the training of AI detection and characterization systems themselves—a use that would demand far more stringent validation against distributional bias and the risk of models learning from their own artifacts.

The deeper lesson of this work may be cultural rather than technical. Endoscopists are trained to trust their eyes, and this study demonstrates, elegantly and a little unnervingly, that the eye can now be fooled by a machine. The same lesson is emerging across imaging: in a recent multicenter study, neither radiologists nor multimodal language models could reliably distinguish language model-generated radiographs from authentic ones, prompting calls to train clinicians to recognize synthetic images. 10 That indistinguishability is both an opportunity and a caution. The opportunity is a renewable supply of teaching material unshackled from privacy constraints; the caution is twofold—that we must verify educational benefit and representativeness before synthetic images shape how the next generation learns to see, and that the realism which makes them useful for teaching also lowers the barrier to misuse. Sodmann and colleagues have cleared the first hurdle—showing that the images look real. The harder and more important work, showing that they teach, lies ahead.
